# On the Lawson–Lim means and Karcher mean for positive invertible operators

**DOI:** 10.1186/s13660-018-1817-5

**Published:** 2018-09-05

**Authors:** Wenshi Liao, Pujun Long, Zemin Ren, Junliang Wu

**Affiliations:** 1 College of Mathematics and Physics, Chongqing University of Science and Technology, Chongqing, China; 2 College of Mathematics and Statistics, Chongqing University, Chongqing, China

**Keywords:** 15A45, 47A63, 47A64, Karcher mean, Lawson–Lim geometric mean, Ando–Li–Mathias geometric mean, Kantorovich constant

## Abstract

This note aims to generalize the reverse weighted arithmetic–geometric mean inequality of *n* positive invertible operators due to Lawson and Lim. In addition, we make comparisons between the weighted Karcher mean and Lawson–Lim geometric mean for higher powers.

## Introduction

Let $B(\mathcal{H})$ be the $C^{*}$-algebra of all bounded linear operators on a complex separable Hilbert space $\mathcal{H}$. $B(\mathcal{H})^{+}$ stands for the set of positive elements in $B(\mathcal{H})$. A linear map Φ: $B(\mathcal{H})\rightarrow B(\mathcal{H})$ is said to be positive (strictly positive) if $\Phi (A)\ge0$ ($\Phi(A)>0$) whenever $A\ge0$ ($A>0$). A positive linear map is said to be normalized (unital) if $\Phi(I)=I$. Note that a positive linear map Φ is monotone in the sense that $A\le B$ implies $\Phi(A)\le\Phi(B)$. $\mathbb{P}$ stands for the convex cone of positive invertible operators. $\Delta_{n}$ denotes the simplex of positive probability vectors in $\mathbb{R}^{n}$ convexly spanned by the unit coordinate vectors. $\|\cdot\|$ and $|\!\|\cdot|\!\|$ denote the operator norm and the unitarily invariant norm, respectively.

Since the pioneering papers of Pusz and Woronowicz [18], Ando [1], and Kubo and Ando [11], an extensive theory of two-variable geometric mean has sprung up for positive operators: For two positive operators *A* and *B*, the operator geometric mean is defined by $A\sharp B:=A^{\frac{1}{2}}(A^{-\frac{1}{2}}BA^{-\frac{1}{2}})^{\frac {1}{2}} A^{\frac{1}{2}}$ for $A>0$. But the *n*-variable case for $n> 2$ was a long standing problem and many authors studied the geometric mean of *n*-variable.

In 2004, Ando et al. [2] succeeded in the formulation of the geometric mean for *n* positive definite matrices, and they showed that it satisfies ten important properties.

### Definition 1.1

(Ando–Li–Mathias geometric mean [2])

Let $A_{i}$ $(i=1,2,\ldots,n)$ be positive definite matrices. Then the geometric mean $G_{\mathrm{ALM}}(A_{1},A_{2},\ldots,A_{n})$ is defined by induction as follows:

(i) $G_{\mathrm{ALM}}(A_{1},A_{2})=A_{1}\#A_{2}$.
(ii) Assume that the geometric mean of any $n-1$-tuple of operators is defined. Let
  $$G_{\mathrm{ALM}}\bigl((A_{j})_{j\neq i}\bigr)=G_{\mathrm{ALM}}(A_{1}, \ldots,A_{i-1},A_{i+1},\ldots,A_{n}), $$

and let the sequences $\{A^{(r)}_{i}\}^{\infty}_{r=0}$ be $A^{(0)}_{i}=A_{i}$ and $A^{(r)}_{i} =G_{\mathrm{ALM}}((A^{(r-1)}_{j})_{j\neq i})$. If there exists $\lim_{r\rightarrow\infty}A^{(r)}_{i}$, and it does not depend on *i*, then the geometric mean of *n*-matrices is defined as

$$\lim_{r\rightarrow\infty}A^{(r)}_{i}=G_{\mathrm{ALM}}(A_{1},A_{2}, \ldots,A_{n}). $$

In [20], Yamazaki pointed out that the definition of the geometric mean by Ando, Li and Mathias can be extended to Hilbert space operators. Lawson and Lim [12] established a definition of the weighted version of the Ando–Li–Mathias geometric mean for *n* positive operators, we call it *Lawson–Lim geometric mean* $G[n,t](A_{1},A_{2},\ldots ,A_{n})$; see [12] for more details. In particular, $G[n,\frac{1}{2}]$ for $t=\frac{1}{2}$ is the Ando-Li-Mathias geometric mean. Similarly, the weighted arithmetic mean is defined as follows:

$$A[n,t](A_{1},A_{2},\ldots,A_{n})=t[n]_{1}A_{1}+t[n]_{2}A_{2}+ \cdots+t[n]_{n}A_{n}, $$

where $t[n]_{i}\ge0$ for all $i=1,2,\ldots,n$ with $\sum_{i=1}^{n} t[n]_{i}=1$. Also, the weighted harmonic mean $H[n,t](A_{1},A_{2},\ldots,A_{n})$ is defined as

$$H[n,t](A_{1},A_{2},\ldots,A_{n})= \bigl(t[n]_{1}A_{1}^{-1}+t[n]_{2}A_{2}^{-1}+ \cdots+t[n]_{n}A_{n}^{-1} \bigr)^{-1}. $$

Note that the coefficient $\{t[n]_{i}\}$ depends on *n* and *t* only; see [6, 19] for more details.

Moreover, the weighted arithmetic–geometric-harmonic mean inequalities holds:

1.1 $$ H[n,t](A_{1},A_{2},\ldots,A_{n})\le G[n,t](A_{1},A_{2},\ldots,A_{n}) \le A[n,t](A_{1},A_{2},\ldots,A_{n}). $$

Since then, another approach to generalizing the geometric mean to *n*-variables, depending on Riemannian trace metric, was the Karcher mean, which was studied by many researchers; see [13, 14] and the references therein. Let $\mathbb{A}=A_{1}, A_{2},\ldots,A_{n}\in\mathbb{P}^{n}$ and $\omega =(w_{1},w_{2},\ldots, w_{n})\in\Delta_{n}$. By computing appropriate derivatives as in [3], the *ω*-weighted *Karcher mean* of $\mathbb{A}$, denoted by $G_{K}(\omega;\mathbb{A})$, coincides with the unique positive definite solution of the Karcher equation

1.2 $$ \sum_{i=1}^{n}w_{i} \log\bigl(X^{\frac{1}{2}}A_{i}^{-1}X^{-\frac{1}{2}}\bigr)=0. $$

In the case of two operators, $A_{1}, A_{2}\in\mathbb{P}$, the Karcher mean coincides with the weighted geometric mean $A_{1}\sharp_{t} A_{2} =A_{1}^{\frac{1}{2}}(A_{1}^{-\frac{1}{2}}A_{2}A_{1}^{-\frac{1}{2}})^{t} A_{1}^{\frac{1}{2}}$. From (1.2), the Karcher mean satisfies the self-duality $G_{K}(\omega;\mathbb{A})=G_{K}(\omega;\mathbb{A}^{-1})^{-1}$, where $\mathbb{A}^{-1}=(A^{-1}_{1},A^{-1}_{2},\ldots,A^{-1}_{n})$.

## Weighted arithmetic and geometric means due to Lawson and Lim

In 2006, Yamazaki [20] obtained a converse of the arithmetic–geometric mean inequality of *n*-operators via Kantorovich constant. Soon after, Fujii el al. [6] also proved a stronger reverse inequality of the weighted arithmetic and geometric means due to Lawson and Lim of *n*-operators by the Kantorovich inequality.

In this section, we present the higher-power reverse inequalities of the weighted arithmetic and geometric means due to Lawson and Lim of *n*-operators, and several complements of the weighted geometric mean for *n*-variables have been established.

### Lemma 2.1

([4, 5])

*Let* $A, B\ge0$. *Then the following inequality holds*:

2.1 $$ \Vert AB \Vert \le\frac{1}{4} \Vert A+B \Vert ^{2}. $$

### Remark 2.1

Drury [5] recently established a remarkable improvement of (2.1) when $A, B$ are, moreover, compact. More precisely, if $A, B\geq0$ are compact, then there exists an isometry *U* such that

2.2 $$ U \vert AB \vert U^{*}\le\frac{1}{4}(A+B)^{2}. $$

### Lemma 2.2

([3, p. 28])

*Let* $A, B\ge0$. *Then*, *for* $1\le r<+\infty$,

2.3 $$ \bigl\Vert A^{r}+B^{r} \bigr\Vert \le \bigl\Vert (A+B)^{r} \bigr\Vert . $$

It is well known that $\|A\|\le1$ is equivalent to $A^{*}A\le I$. This fact plays an important role in the proofs of the theorems.

### Theorem 2.1

*For any positive integer* $n\ge2$, *let* $A_{1}, A_{2}, \ldots, A_{n}$ *be positive invertible operators on a Hilbert space* $\mathcal{H}$ *such that* $m\le A_{i}\le M$ *for* $i=1,2,\ldots,n$ *and some scalars* $0< m< M$. *Then*, *for* $p\ge2$,

2.4 $$ A[n,t](A_{1},\ldots, A_{n})^{p}\le \frac{(m+M)^{2p}}{16m^{p}M^{p}}G[n,t](A_{1},\ldots, A_{n})^{p}. $$

### Proof

Let a map $\Psi: \mathcal{B}(\mathcal{H})\oplus\cdots\oplus\mathcal {B}(\mathcal{H}) \mapsto\mathcal{B}(\mathcal{H})$ be defined by

$$\Psi( A_{1}\oplus\cdots\oplus A_{n} )= t[n]_{1}A_{1}+ \cdots+t[n]_{n}A_{n}. $$

Then Ψ is a positive linear map such that $\Psi(I)=I$. The condition $0< m\le A_{i}\le M$ for $i=1,2,\ldots,n$ implies that

2.5 $$ m\le\Psi( A_{1}\oplus\cdots\oplus A_{n} ) \le M. $$

By (2.3) in [16], we have

$$\Psi( A_{1}\oplus\cdots\oplus A_{n} )+ Mm\Psi\bigl( A_{1}^{-1}\oplus\cdots\oplus A_{n}^{-1} \bigr) \le M+m. $$

Thus,

2.6 $$ A[n,t](A_{1},\ldots, A_{n})+Mm A[n,t] \bigl(A_{1}^{-1},\ldots, A_{n}^{-1}\bigr) \le m+M. $$

On the other hand, by computing, we deduce

$$\begin{aligned} &\bigl\Vert M^{\frac{p}{2}}m^{\frac{p}{2}}A[n,t](A_{1}, \ldots, A_{n})^{\frac{p}{2}}G[n,t](A_{1},\ldots, A_{n})^{-\frac{p}{2}} \bigr\Vert \\ &\quad\le\frac{1}{4} \bigl\Vert A[n,t](A_{1},\ldots, A_{n})^{\frac{p}{2}}+M^{\frac{p}{2}}m^{\frac{p}{2}}G[n,t](A_{1},\ldots, A_{n})^{-\frac{p}{2}} \bigr\Vert ^{2} \quad\bigl(\text{by } (2.1)\bigr) \\ &\quad\le\frac{1}{4} \bigl\Vert A[n,t](A_{1},\ldots, A_{n})+MmG[n,t](A_{1},\ldots, A_{n})^{-1} \bigr\Vert ^{p} \\ &\qquad \biggl(\text{by } (2.3) \text{ and } \frac{p}{2}\ge1\biggr) \\ &\quad= \frac{1}{4} \bigl\Vert A[n,t](A_{1},\ldots, A_{n})+MmG[n,t]\bigl(A_{1}^{-1},\ldots, A_{n}^{-1}\bigr) \bigr\Vert ^{p} \\ &\quad\le\frac{1}{4} \bigl\Vert A[n,t](A_{1},\ldots, A_{n})+MmA[n,t]\bigl(A_{1}^{-1},\ldots, A_{n}^{-1}\bigr) \bigr\Vert ^{p} \quad\bigl(\text{by } (1.1)\bigr) \\ &\quad\le\frac{(M+m)^{p}}{4}\quad \bigl(\text{by } (2.6)\bigr). \end{aligned}$$

The equality above follows from the self-duality of the geometric mean (see [2, 6, 20]). □

Taking $p=2$, (2.4) implies the following corollary.

### Corollary 2.1

*For any positive integer* $n\ge2$, *let* $A_{1}, A_{2}, \ldots, A_{n}$ *be positive invertible operators on a Hilbert space* $\mathcal{H}$ *such that* $m\le A_{i}\le M$ *for* $i=1,2,\ldots,n$ *and some scalars* $0< m< M$. *Then*

2.7 $$ A[n,t](A_{1},\ldots, A_{n})^{2}\le \frac{(m+M)^{4}}{16m^{2}M^{2}}G[n,t](A_{1},\ldots, A_{n})^{2}. $$

Note that if $t=\frac{1}{2}$, the inequality (2.7) reduces to Lin’s result (see [15, Theorem 3.2]). By the Löewner–Heinz inequality, we can easily get Theorem 7 in [6] from (2.7).

### Theorem 2.2

*For any positive integer* $n\ge2$, *let* $A_{1}, A_{2}, \ldots, A_{n}$ *be positive invertible operators on a Hilbert space* $\mathcal{H}$ *such that* $m\le A_{i}\le M$ *for* $i=1,2,\ldots,n$ *and some scalars* $0< m< M$. *Then*, *for* $1<\alpha\le2$ *and* $p\ge2\alpha$,

2.8 $$ A[n,t](A_{1},\ldots, A_{n})^{p}\le \frac{(k^{\frac{\alpha}{2}}(M^{\alpha}+m^{\alpha}))^{\frac{2p}{\alpha }}}{16M^{p}m^{p}}G[n,t](A_{1},\ldots, A_{n})^{p}, $$

*where* $k=\frac{(m+M)^{2}}{4mM}$.

### Proof

Let a map $\Psi: \mathcal{B}(\mathcal{H})\oplus\cdots\oplus\mathcal {B}(\mathcal{H}) \mapsto\mathcal{B}(\mathcal{H})$ be defined as in the proof of Theorem 2.1. By (2.5) and the Löewner–Heinz inequality, we have

$$m^{\alpha}\le\Psi^{\alpha}( A_{1}\oplus\cdots\oplus A_{n} ) \le M^{\alpha}, $$

that is,

$$m^{\alpha}\le A[n,t](A_{1},\ldots, A_{n})^{\alpha}\le M^{\alpha}. $$

By (2.3) in [16], we have

2.9 $$ A[n,t](A_{1},\ldots, A_{n})^{\alpha}+M^{\alpha}m^{\alpha}A[n,t](A_{1},\ldots, A_{n})^{-\alpha} \le m^{\alpha}+M^{\alpha}. $$

On the other hand, by (2.7),

2.10 $$ k^{-\alpha}G[n,t](A_{1},\ldots, A_{n})^{-\alpha}\le A[n,t](A_{1},\ldots, A_{n})^{-\alpha}. $$

By computing, we deduce

$$\begin{aligned} & \bigl\Vert k^{-\frac{p}{2}}m^{\frac{p}{2}}M^{\frac{p}{2}}A[n,t](A_{1}, \ldots, A_{n})^{\frac{p}{2}}G[n,t](A_{1},\ldots, A_{n})^{-\frac{p}{2}} \bigr\Vert \\ &\quad\le\frac{1}{4} \bigl\Vert A[n,t](A_{1},\ldots, A_{n})^{\frac{p}{2}}+k^{-\frac{p}{2}}m^{\frac{p}{2}}M^{\frac{p}{2}}G[n,t](A_{1}, \ldots, A_{n})^{-\frac{p}{2}} \bigr\Vert ^{2} \quad\bigl(\text{by } (2.1)\bigr) \\ &\quad\le\frac{1}{4} \bigl\Vert \bigl(A[n,t](A_{1},\ldots, A_{n})^{\alpha}+k^{-\alpha}m^{\alpha}M^{\alpha}G[n,t](A_{1}, \ldots, A_{n})^{-\alpha}\bigr)^{\frac{p}{2\alpha}} \bigr\Vert ^{2} \\ &\qquad \biggl(\text{by } (2.3) \text{ and } \frac{p}{2}\ge1\biggr) \\ &\quad= \frac{1}{4} \bigl\Vert A[n,t](A_{1},\ldots, A_{n})^{\alpha}+k^{-\alpha}m^{\alpha}M^{\alpha}G[n,t](A_{1}, \ldots, A_{n})^{-\alpha} \bigr\Vert ^{\frac{p}{\alpha}} \\ &\quad\le\frac{1}{4} \bigl\Vert A[n,t](A_{1},\ldots, A_{n})^{\alpha}+m^{\alpha}M^{\alpha}A[n,t](A_{1}, \ldots, A_{n})^{-\alpha} \bigr\Vert ^{p} \quad\bigl(\text{by } (2.10)\bigr) \\ &\quad\le\frac{(M^{\alpha}+m^{\alpha})^{p}}{4} \quad\bigl(\text{by } (2.9)\bigr). \end{aligned}$$

We obtain the desired result. □

Putting $\alpha=2$ in the inequality (2.8) implies the following.

### Corollary 2.2

*Under the same conditions as in Theorem *2.2, *then*, *for* $p\ge4$,

2.11 $$ A[n,t](A_{1},\ldots, A_{n})^{p}\le \frac{(k(M^{2}+m^{2}))^{p}}{16M^{p}m^{p}}G[n,t](A_{1},\ldots, A_{n})^{p}. $$

### Remark 2.2

When $\frac{M}{m}\le2+\sqrt{3}$, we have $\frac {(m+M)^{2p}}{16m^{p}M^{p}}\ge\frac{(k(M^{2}+m^{2}))^{p}}{16M^{p}m^{p}}$, it is easy to see that (2.11) is sharper than (2.4) for $p\ge4$.

Next, we show the complements of the weighted geometric mean due to Lawson and Lim by virtue of the following lemma (see Corollary 2.12 in [10]) and we will generalize Lemma 2.8 and Lemma 2.9 in [19] in two following theorems.

### Lemma 2.3

([10])

*For any integer* $n\ge2$, *let* $A_{1},A_{2},\ldots,A_{n}$ *be positive invertible operators in* $\mathbb{P}$ *such that* $m\le A_{i}\le M$ *for all* $i=1,2,\ldots,n$ *and some scalars* $0< m\le M$. *Then*

2.12 $$ A[n,t]\bigl(A^{p}_{1},\ldots,A^{p}_{n} \bigr)\le K(m,M,p)A[n,t](A_{1},\ldots, A_{n})^{p}\quad \textit{for all }p\ge1, $$

*where* $K(m,M,p)=\frac{(p-1)^{p-1}}{p^{p}}\frac {(M^{p}-m^{p})^{p}}{(M-m)(mM^{p}-Mm^{p})^{p-1}}$ *is the generalized Kantorovich constant*.

### Proof

By Corollary 2.6 in [17],

$$\Phi\bigl(A^{p}\bigr)\le K(m,M,p)\Phi(A)^{p} \quad\text{for all } p\ge1. $$

Let the map $\Phi: \mathcal{B}(\mathcal{H})\oplus\cdots\oplus\mathcal {B}(\mathcal{H}) \mapsto\mathcal{B}(\mathcal{H})$ be defined as Ψ in the proof of Theorem 2.1. Then, for $p\ge1$,

$$A[n,t]\bigl(A^{p}_{1},\ldots,A^{p}_{n} \bigr)\le K(m,M,p)A[n,t](A_{1},\ldots, A_{n})^{p}. $$

 □

### Theorem 2.3

*For any integer* $n\ge2$, *let* $A_{1},A_{2},\ldots,A_{n}$ *be positive invertible operators in* $\mathbb{P}$ *such that* $m\le A_{i}\le M $ *for all* $i=1,2,\ldots,n$ *and some scalars* $0< m\le M$. *Then*

$$G[n,t]\bigl(A^{p}_{1},\ldots,A^{p}_{n} \bigr)\le K(m,M,p)\frac{(m+M)^{2p}}{4^{p}m^{p}M^{p}}G[n,t](A_{1},\ldots, A_{n})^{p}\quad \textit{for all }1< p\le2 $$

*and*

$$G[n,t]\bigl(A^{p}_{1},\ldots,A^{p}_{n} \bigr)\le K(m,M,p)\frac{(m+M)^{2p}}{16m^{p}M^{p}}G[n,t](A_{1},\ldots, A_{n})^{p}\quad \textit{for all }p\ge2. $$

### Proof

By the arithmetic–geometric mean inequality and (2.12), it follows that

2.13 $$\begin{aligned} G[n,t]\bigl(A^{p}_{1}, \ldots,A^{p}_{n}\bigr)&\le A[n,t]\bigl(A^{p}_{1}, \ldots,A^{p}_{n}\bigr) \\ &\le K(m,M,p)A[n,t](A_{1},\ldots, A_{n})^{p} \quad\text{for } p\ge1. \end{aligned}$$

For $p\in(1,2]$, it follows from (2.7) and the Löewner–Heinz inequality that

$$A[n,t](A_{1},\ldots, A_{n})^{p}\le\biggl( \frac{(m+M)^{2}}{4mM} \biggr)^{p}G[n,t](A_{1},\ldots, A_{n})^{p}. $$

Combining the inequalities above, we have

$$G[n,t]\bigl(A^{p}_{1},\ldots,A^{p}_{n} \bigr)\le K(m,M,p)\frac{(m+M)^{2p}}{4^{p}m^{p}M^{p}}G[n,t](A_{1},\ldots, A_{n})^{p}. $$

For $p\in[2,\infty)$, from (2.4) and (2.13), we obtain

$$G[n,t]\bigl(A^{p}_{1},\ldots,A^{p}_{n} \bigr)\le K(m,M,p)\frac{(m+M)^{2p}}{16m^{p}M^{p}}G[n,t](A_{1},\ldots, A_{n})^{p}. $$

 □

In the following remark, we present the case of $p\ge1$ for the Ando–Li–Mathias geometric mean.

### Remark 2.3

Let $t=\frac{1}{2}$ in Theorem 2.3. Then

$$G_{\mathrm{ALM}}\bigl(A^{p}_{1},\ldots,A^{p}_{n} \bigr)\le K(m,M,p)\frac{(m+M)^{2p}}{4^{p}m^{p}M^{p}}G_{\mathrm {ALM}}(A_{1},\ldots, A_{n})^{p} \quad\text{for all }1< p\le2 $$

and

$$G_{\mathrm{ALM}}\bigl(A^{p}_{1},\ldots,A^{p}_{n} \bigr)\le K(m,M,p)\frac{(m+M)^{2p}}{16m^{p}M^{p}}G_{\mathrm{ALM}}(A_{1},\ldots, A_{n})^{p} \quad\text{for all }p\ge2. $$

### Theorem 2.4

*For any integer* $n\ge2$, *let* $A_{1},A_{2},\ldots,A_{n}$ *be positive invertible operators in* $\mathbb{P}$ *such that* $m\le A_{i}\le M $ *for all* $i=1,2,\ldots,n$ *and some scalars* $0< m\le M$. *Then*

$$G[n,t]\bigl(A^{p}_{1},\ldots,A^{p}_{n} \bigr)^{\frac{1}{p}}\le K \biggl(m^{q},M^{q}, \frac{p}{q} \biggr)^{\frac{1}{p}} \biggl(\frac{(m^{q}+M^{q})^{2}}{4m^{q}M^{q}} \biggr)^{\frac{1}{q}}G[n,t]\bigl(A^{q}_{1}, \ldots,A^{q}_{n}\bigr)^{\frac{1}{q}} $$

*for all* $1<\frac{p}{q}\le2$ *and* $p\ge1$, *and*

$$G[n,t]\bigl(A^{p}_{1},\ldots,A^{p}_{n} \bigr)^{\frac{1}{p}}\le4^{-\frac{2}{p}} K \biggl(m^{q},M^{q}, \frac{p}{q} \biggr)^{\frac{1}{p}} \biggl(\frac{(m^{q}+M^{q})^{2}}{m^{q}M^{q}} \biggr)^{\frac{1}{q}}G[n,t]\bigl(A^{q}_{1}, \ldots,A^{q}_{n}\bigr)^{\frac{1}{q}} $$

*for all* $\frac{p}{q}\ge2$ *and* $p\ge1$.

### Proof

For each $0< q\le p$, it follows from the arithmetic–geometric mean inequality (1.1) and (2.12) that

2.14 $$\begin{aligned} G[n,t]\bigl(A^{p}_{1}, \ldots,A^{p}_{n}\bigr)&\le A[n,t]\bigl(A^{p}_{1}, \ldots,A^{p}_{n}\bigr) \\ &= A[n,t]\bigl(\bigl(A^{q}_{1}\bigr)^{\frac{p}{q}}, \ldots,\bigl(A^{q}_{n}\bigr)^{\frac{p}{q}}\bigr) \\ &\le K \biggl(m^{q},M^{q},\frac{p}{q} \biggr)A[n,t] \bigl(A^{q}_{1},\ldots, A^{q}_{n} \bigr)^{\frac{p}{q}}. \end{aligned}$$

On the other hand, for $1<\frac{p}{q}\le2$, from (2.7) and $m^{q}\le A_{i}^{q}\le M^{q}$, it follows that

$$A[n,t]\bigl(A^{q}_{1},\ldots, A^{q}_{n} \bigr)^{\frac{p}{q}}\le\biggl(\frac{(m^{q}+M^{q})^{2}}{4m^{q}M^{q}} \biggr)^{\frac{p}{q}}G[n,t]\bigl(A^{q}_{1},\ldots,A^{q}_{n} \bigr)^{\frac{p}{q}}. $$

Combining the two inequalities above, we obtain

$$G[n,t]\bigl(A^{p}_{1},\ldots,A^{p}_{n} \bigr)\le K \biggl(m^{q},M^{q},\frac{p}{q} \biggr) \biggl(\frac{(m^{q}+M^{q})^{2}}{4m^{q}M^{q}} \biggr)^{\frac{p}{q}}G[n,t]\bigl(A^{q}_{1}, \ldots,A^{q}_{n}\bigr)^{\frac{p}{q}}. $$

By the Löewner–Heinz inequality and $p\ge1$, it follows that

$$G[n,t]\bigl(A^{p}_{1},\ldots,A^{p}_{n} \bigr)^{\frac{1}{p}}\le K \biggl(m^{q},M^{q}, \frac{p}{q} \biggr)^{\frac{1}{p}} \biggl(\frac{(m^{q}+M^{q})^{2}}{4m^{q}M^{q}} \biggr)^{\frac{1}{q}}G[n,t]\bigl(A^{q}_{1}, \ldots,A^{q}_{n}\bigr)^{\frac{1}{q}}. $$

Similarly, for all $\frac{p}{q}\ge2$, from (2.4) we have

$$A[n,t]\bigl(A^{q}_{1},\ldots, A^{q}_{n} \bigr)^{\frac{p}{q}}\le4^{-2} \biggl(\frac{(m^{q}+M^{q})^{2}}{m^{q}M^{q}} \biggr)^{\frac{p}{q}}G[n,t]\bigl(A^{q}_{1}, \ldots,A^{q}_{n}\bigr)^{\frac{p}{q}}. $$

Combining with (2.14), we obtain

$$G[n,t]\bigl(A^{p}_{1},\ldots,A^{p}_{n} \bigr)\le4^{-2}K \biggl(m^{q},M^{q}, \frac{p}{q} \biggr) \biggl(\frac{(m^{q}+M^{q})^{2}}{m^{q}M^{q}} \biggr)^{\frac{p}{q}}G[n,t]\bigl(A^{q}_{1},\ldots,A^{q}_{n} \bigr)^{\frac{p}{q}}. $$

It follows from $p\ge1$ that

$$G[n,t]\bigl(A^{p}_{1},\ldots,A^{p}_{n} \bigr)^{\frac{1}{p}}\le4^{-\frac{2}{p}}K \biggl(m^{q},M^{q}, \frac{p}{q} \biggr)^{\frac{1}{p}} \biggl(\frac{(m^{q}+M^{q})^{2}}{m^{q}M^{q}} \biggr)^{\frac{1}{q}}G[n,t]\bigl(A^{q}_{1}, \ldots,A^{q}_{n}\bigr)^{\frac{1}{q}}. $$

This completes the proof. □

## Comparisons between the weighted Karcher mean and the Lawson–Lim geometric mean

In the final section, we make comparisons between the weighted Karcher mean and the Lawson–Lim geometric mean for higher powers. This is a fascinating work because the order relation can be preserved between higher-power operators by the Kantorovich constant.

### Lemma 3.1

([8])

*Let* $0< m\le A\le M$ *and* $A\le B$. *Then*

$$A^{2}\le\frac{(M+m)^{2}}{4Mm}B^{2}. $$

### Lemma 3.2

([9])

*Let* *A* *and* *B* *be positive invertible operators on a Hilbert space* $\mathcal{H}$ *satisfying* $B\ge A>0$ *and* $0< m\le A\le M$. *Then*

$$\mathrm{K}(m,M,p)B^{p}\ge A^{p} $$

*holds for any* $p\ge1$, *where* $\mathrm{K}(m,M,p)$ *is the generalized Kantorovich constant or the Ky Fan–Furuta constant*.

### Theorem 3.1

*For any positive integer* $n\ge2$, *let* $A_{1}, A_{2}, \ldots, A_{n}$ *be positive invertible operators on a Hilbert space* $\mathcal{H}$ *such that* $m\le A_{i}\le M$ *for* $i=1,2,\ldots,n$ *and some scalars* $0< m< M$. *Then*

3.1 $$\begin{aligned} & G_{K}(\omega,A_{1},\ldots, A_{n})^{p} \\ &\quad\le\mathrm{K}(m,M,p)\frac{(M+m)^{2p}}{4^{p}M^{p}m^{p}}G[n,t](A_{1}, \ldots, A_{n})^{p} \quad\textit{for all } 1\le p\le2 \end{aligned}$$

*and*

3.2 $$ G_{K}(\omega,A_{1},\ldots, A_{n})^{p}\le\mathrm{K}(m,M,p)\frac{(M+m)^{2p}}{16M^{p}m^{p}}G[n,t](A_{1}, \ldots, A_{n})^{p} \quad\textit{for all }p\ge2. $$

### Proof

By the Löewner–Heinz inequality and (2.7), we have

3.3 $$ A[n,t](A_{1},\ldots, A_{n})^{p}\le \frac{(M+m)^{2p}}{4^{p}M^{p}m^{p}}G[n,t](A_{1},\ldots, A_{n})^{p} \quad\text{for all } 1\le p\le2. $$

It follows from Lemma 3.2 and (3.3) that

$$\begin{aligned} G_{K}(\omega,A_{1},\ldots, A_{n})^{p}& \le\mathrm{K}(m,M,p)A[n,t](A_{1},\ldots, A_{n})^{p} \\ &\le\mathrm{K}(m,M,p)\frac{(M+m)^{2p}}{4^{p}M^{p}m^{p}}G[n,t](A_{1},\ldots, A_{n})^{p}. \end{aligned}$$

The inequality (3.2) follows from Lemma 3.2 and (2.4) that

$$\begin{aligned} G_{K}(\omega,A_{1},\ldots, A_{n})^{p}& \le\mathrm{K}(m,M,p)A[n,t](A_{1},\ldots, A_{n})^{p} \\ &\le\mathrm{K}(m,M,p)\frac{(M+m)^{2p}}{16M^{p}m^{p}}G[n,t](A_{1},\ldots, A_{n})^{p}. \end{aligned}$$

 □

### Remark 3.1

When $p=1, t=\frac{1}{2}$, the inequality (3.1) reduces to the first inequality of Theorem 5.1 in [7].
